# Single-walled carbon nanotubes as a photo-thermo-acoustic cancer theranostic agent: theory and proof of the concept experiment

**DOI:** 10.1038/s41598-020-79238-6

**Published:** 2020-12-17

**Authors:** L. Golubewa, I. Timoshchenko, O. Romanov, R. Karpicz, T. Kulahava, D. Rutkauskas, M. Shuba, A. Dementjev, Yu. Svirko, P. Kuzhir

**Affiliations:** 1 Center for Physical Sciences and Technology, Sauletekio Ave. 3, 10257 Vilnius, Lithuania; 2 Institute for Nuclear Problems, Belarusian State University, Bobruiskaya 11, 220006 Minsk, Belarus; 3 Belarusian State University, Nezavisimosti Ave. 4, 220030 Minsk, Belarus; 4 Tomsk State University, Lenin Ave. 36, Tomsk, Russia 634050; 5 Institute of Photonics, University of Eastern Finland, Yliopistokatu 7, 80100 Joensuu, Finland

**Keywords:** Nanoscale materials, Nanobiotechnology, Nanotechnology in cancer, Biophotonics, Photoacoustics, Biophysics, Cancer

## Abstract

Theranostics is the emerging field of medicine that uniquely combines diagnostic techniques and active agents to diagnose and treat medical conditions simultaneously or sequentially. Finding a theranostic agent capable to cure the affected cells and being safe for the healthy ones is the key for successful treatment. Here, we demonstrate that agglomerated single-walled carbon nanotubes (SWCNTs) are promising theranostic agent that enables photo-activated ‘cold’ destruction of the cancer cells keeping their environment alive. The absorption of picosecond pulses by SWCNT agglomerates results in the mechanical (due to photoacoustic effect) rather than photothermal cancer cell destruction, which was visualized by micro-Raman and ultrafast near-infrared CARS. The developed theoretical model allows us to distinguish photothermal, photoacoustic, and photothermoacoustic regimes of the cancer cell destruction, and also to optimize SWCNT-based theranostics recipe.

## Introduction

Lasers are widely used in biomedicine because the irradiation of living cells and tissues with coherent light beams may result in a variety of effects. Among the most examined ones are wound healing, tissue coagulation and vaporization, tissue ablation, and necrosis^1^. However, for the tissue embedded with micro- or nanoparticles (NPs) having the sharp absorption lines in the so-called therapeutic window of 650–1350 nm^2^, one can employ photothermal^3^, photothermoacoustic^4^, and photoacoustic^5^ effects for treatment. Owing to their ability to target tumor tissue, as well as their capacity to be manipulated by light, NPs are perfectly suitable for theranostic cancer screening, staging, and treatment^6,7^ using light in the near-infrared (NIR) tissue transparency window^2^.

Photothermal effect (or hyperthermia) is observed when light absorption in NPs results in local overheating of the surrounding tissue^8^ that can lead to protein denaturation, cell membrane lysis, and cell death. Although the temperature needed to destroy cancer cells in vitro is as high as 70–80°C^9^, even lower temperatures can be used to facilitate the release of therapeutic agents carried by NPs^8^. Similar to the Photothermal effect, Photothermoacoustic and Photoacoustic effects manifest themselves as the generation of acoustic waves in the NP’s surrounding tissue^10^ due to the conversion of the absorbed light energy into heat inside a NP. In the Photothermoacoustic effect, the NIR absorption by the NP leads to the temperature increase up to thousands of degrees, the surrounding aqueous medium evaporates forming—in the nanosecond time scale—a gas bubble and leading to an acoustic perturbation and local fluid flow^11,12^. The Photoacoustic effect occurs when the conversion of the light energy into heat inside the NP leads to its deformation, which produces an acoustic wave in the surrounding medium, rather than forms the vapour bubble. The important advantage of this effect for cancer treatment is that in the photoacoustic regime, no heating of the surrounding medium takes place, while the cancer cell damage can be achieved by the pressure pulse generated by the overheated NP. That is search for the appropriate theranostic agent for cancer treatment implies finding NPs that enable photoacoustic regime in the NIR spectral range.

Single-walled carbon nanotubes (SWCNTs) can be such a theranostics agent because their high NIR-absorptive ability^13^ enables photothermal therapy^14^, which is much more specific and much less aggressive compared to conventional chemo- or radiotherapy. High specificity is, to a large extent, the result of SWCNTs’ bioactivity and cellular uptake^15,16^, which in turn may make them toxic^17^. The latter—along with the lack of solubility in aqueous media^18^ as well as biodistribution and pharmacokinetic issues—significantly suppresses the interest of using SWCNTs in biomedicine. However, recent advances in nanotechnology have enabled the mass production of high-quality single-chirality SWCNTs^19^, which can be gently cut^20^, purified to the almost invisible degree of metal catalyst contamination^21^, and functionalized^22^. This has reopened interest to SWCNTs as cancer theranostics agent^23^ because short (100 nm)^24^ and properly functionalized^25^ CNTs are significantly less toxic than long and bare ones.

The properties of biological materials may be changed considerably by embedding carbon NPs^26^. For example, it has been shown that the addition of 1% of CNTs enhances the thermal conductivity of aqueous suspension^27^. By performing SWCNTs’ functionalization or varying the incubation time one may localize SWCNTs either inside the cancer cell^28^, on the cancer cell membrane^29^, or in the surrounding healthy tissue, for example, when transferring through the blood–brain barrier^28^. SWCNT localization and distribution is a crucial factor determining the efficiency of applied therapies, as different types of the external environment, as well as sizes of agglomerates of dispersed SWCNTs, will influence SWCNT response to NIR irradiation. In the biomedical diagnostics and therapy, either suspension of individual (or bundled) SWCNTs or micron-sized SWCNT agglomerates are used. The latter is also formed as a result of cancer cell endocytosis^4^ in vitro, huge SWCNT clusters are formed due to administration of high doses and local accumulation of SWCNTs in mice tissue *in vivo*^30^.

The aim of this paper is to optimize the SWCNT-based photonic theranostics recipe of detection and cold photodestruction of cancer cells by:

1. developing theory and performing a numerical simulation of the interaction of the NIR light with the SWCNTs aggregated in living cells or accumulated in the intercellular medium, and revealing the conditions of the cold photodestruction regime;
2. experimental demonstration of the cold photodestruction of the glioma C6 cancer cells that have captured the aggregated SWCNTs inside and comparison with laser treatment of the cells surrounded by individual SWCNTs situated in the extracellular medium.

## Results

### Theory: interaction of the laser pulses with spherical SWCNT agglomerate embedded into a cancer cell

**Figure 1:**
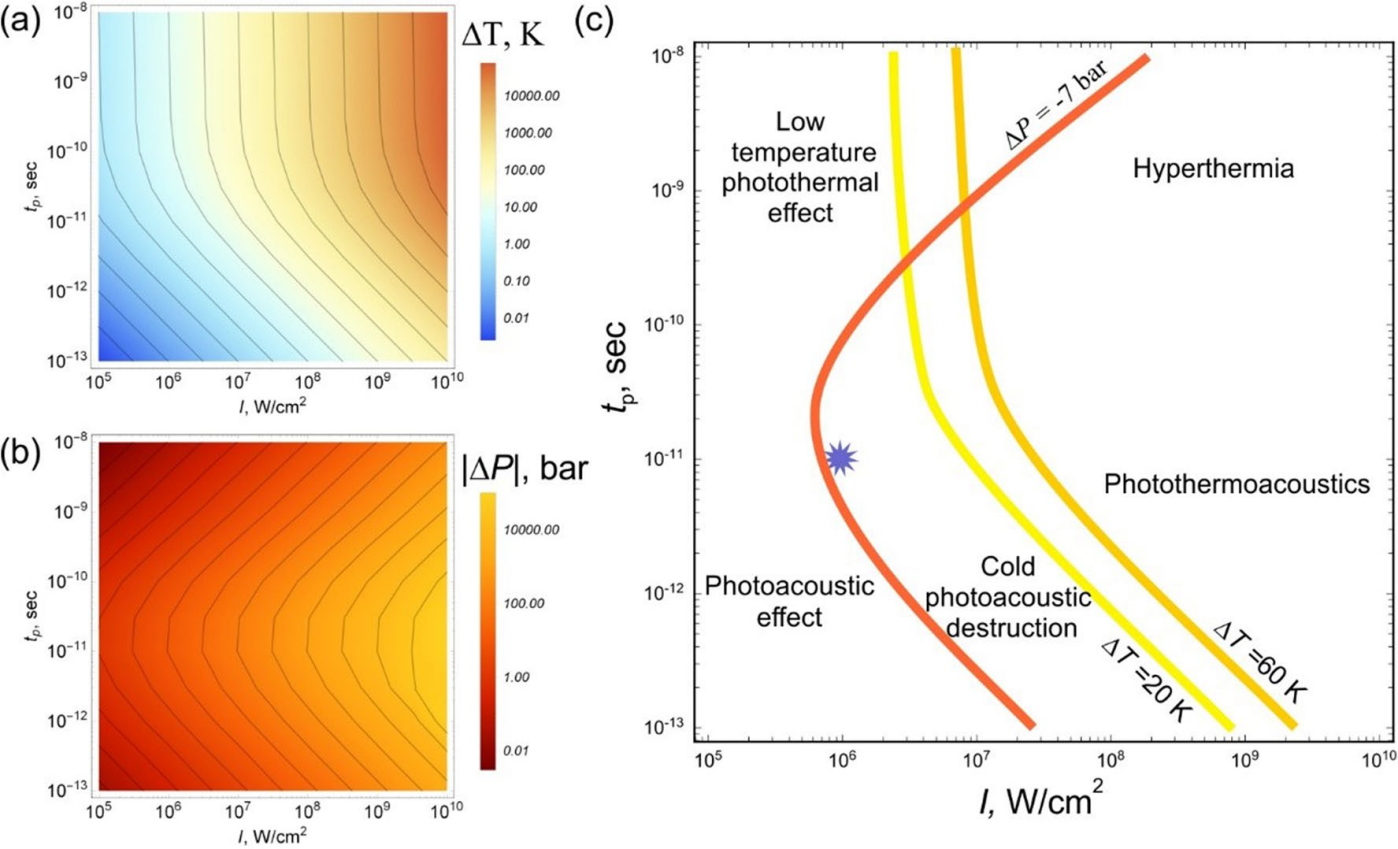
Numerical simulation of the interaction of laser radiation with the SWCNT agglomerate embedded into the living cell. Contour maps of the (**a**) temperature increase and (**b**) negative pressure in the SWCNT agglomerate of *R*_0_ = 1 µm (see Supplementary Information for details) aggregated inside the glioma cell on the pulse duration/intensity plane. (**c**) Isotherms at Δ*T* = 20 K and Δ*T* = 60 K, respectively, and isobar at Δ*P* =  − 0.7 MPa. The star corresponds to the experimental conditions.

In the Supplementary Information, we present the theory of the interaction of light with the spatially inhomogeneous (turbid) medium with embedded NPs, which have a sufficiently low (to ignore the collective effects) concentration and physical properties very different from the host medium. The performed numerical simulation for the spherical SWCNT bundles and individual SWCNTs placed in the living tissue allows us to reveal the conditions when the photothermal, photothermoacoustic and photoacoustic effects occur in the tissue embedded with the SWCNTs. Figure 1a,b shows the contour map of the temperature increase and negative pressure that can be achieved at the center of the spherical SWCNT agglomerate having a radius of *R*_0_ = 1 µm aggregated inside the glioma cell at the initial temperature of 293 K (20 °C). The results of the numerical simulations are summarized in Fig. 1c, which shows characteristic isobar and isotherms in the laser pulse duration-intensity plane.

The yellow line in Fig. 1c presents an isotherm, which corresponds to the agglomerate heating by Δ*T* = 20 K, when the agglomerate temperature of *T* = 313 K (40 °C) achieves the functionality threshold^31^. The brown line in Fig. 1c corresponds to the agglomerate heating by Δ*T* = 60 K, which defines the DNA melting threshold of 353 K (80 °C)^32^ that corresponds to the cell death. In the heating range of 20 K < Δ*T* < 60 K, the temperature-dependent protein denaturation^33^ and inactivation of repair of DNA strand breaks^31^ take place that causes disruption of cellular functioning and may lead to the cell death. Since selective anticancer therapy implies avoiding heating of normal tissue, the heating of the agglomerates below Δ*T* = 20 K is preferable. The dark orange line depicts isobar corresponding to the negative pressure change threshold of 0.7 MPa^34^. Most of biological structures have a sufficiently high resistance to compression and are considerably weaker to tensile stress. If the latter in the negative phase of a pressure wave exceeds the ultimate tensile strength of the medium, there is a high probability of local fragmentation of a cell due to micro-breaks, the formation of microbubbles, and other disturbances in the homogeneity of the structure^35–37^. Acoustic perturbations in the medium can be observed when the pulse duration $t_{{\text{p}}}$ is shorter than time $t_{a} \approx 100\,{\text{ps}}$ of propagation of an acoustic wave through the SWCNT bundle. Photothermal effects prevail at $t_{{\text{p}}} > t_{{\text{a}}}$, because of the acoustic perturbation decays during the irradiation time. Thus, for the pulses shorter than 100 ps the dark orange line separates the parameter domain into photoacoustic non-destructive imaging^38^, for which nanoparticles are often used as contrast agents^39^, and photothermoacoustic regime.

The developed model allows us to distinguish photothermal, photothermoacoustic, and photoacoustic regimes of the interaction of light pulses with SWCNT agglomerates embedded in cytoplasm. This model confirms that the intracellular accumulation of SWCNTs and their agglomeration in micro-sized particles are necessary to implement ‘cold’ photo-induced cancer cell destruction keeping living bare cells intact.

The performed analysis allows us to visualize the cold photoacoustic destruction area (Δ*T* < 20 K and |Δ*P*|> 0.7 MPa), for the SWCNT agglomerates in the living cell. In this area, the irradiation with a laser beam *will not* result in tissue overheating, while the light-induced tensile stress in the SWCNT agglomerate accumulated will destroy the cancer cells. To verify the simulation results we choose for our theranostic experiment laser pulses having an intensity of 2.4 × 10^6^ W/cm^2^ and a duration of 10 ps (see the star in Fig. 1c).

### Experiment: interaction of picosecond laser pulses with glioma cells

We performed an experimental investigation of the interaction of laser radiation with (1) bare glioma cells (as a control sample); (2) glioma cells with intracellularly accumulated and aggregated micron-sized SWCNT agglomerates; and (3) glioma cells placed into the extracellular medium with suspended individual SWCNTs.

**Figure 2:**
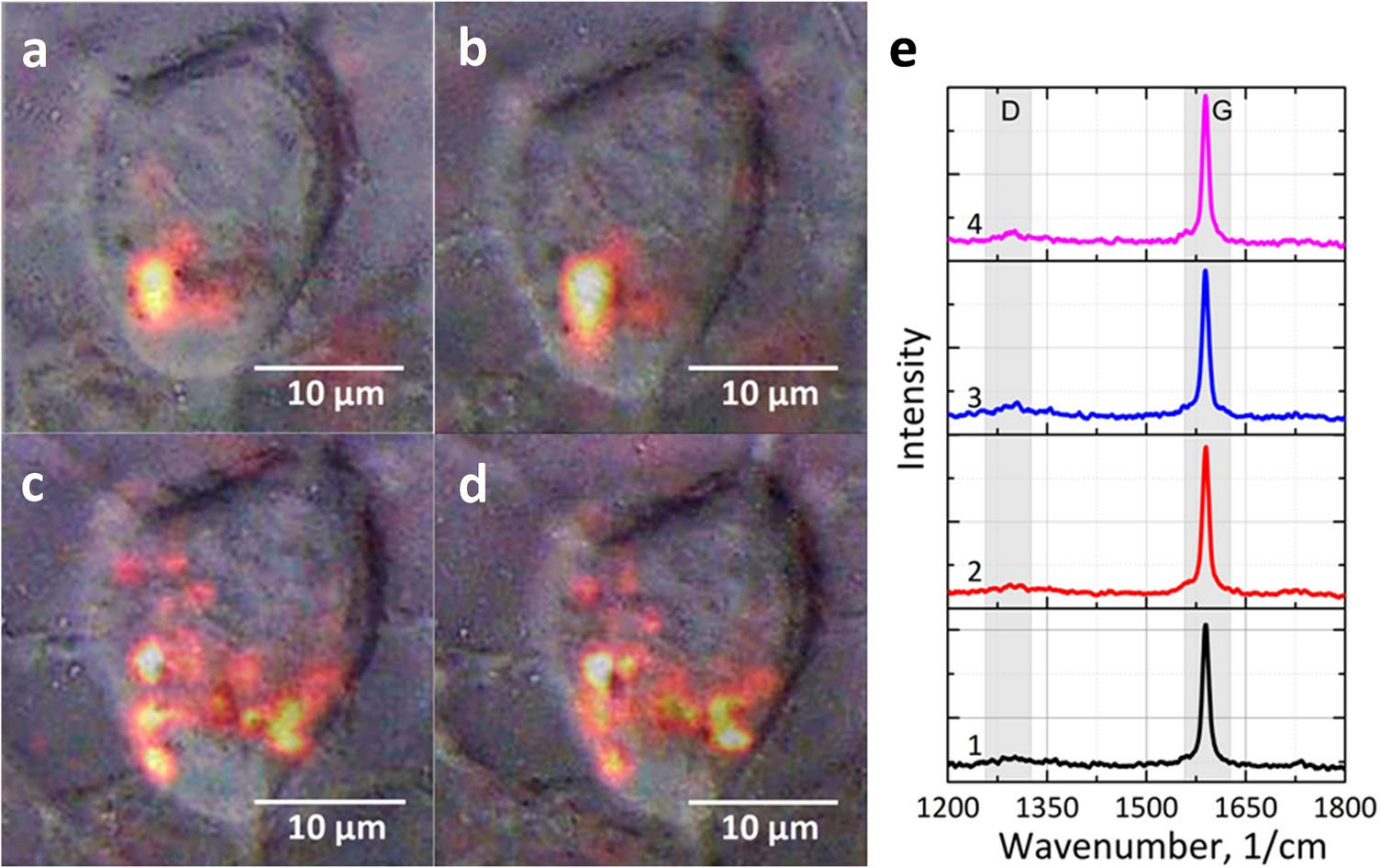
Intracellular accumulation of SWCNTs and structural integrity of C6 glioma cells exposed to continuous laser irradiation with λ_ex_ = 785 nm. (**a**–**d**) images of C6 cells in reflected light superimposed on the map of the Raman G-mode of SWCNTs; (**e**) Raman spectra of SWCNT agglomerates inside the cell. The cell image (**a**) and Raman spectrum (**e-1)** are taken before laser exposure. The cell image (**b**) and Raman spectrum (**e-2**) are taken after irradiation of the cell by a laser beam at the intensity of 7.2 × 10^3^ W/cm^2^ for 10 min. The cell images (**c**,**d**) and Raman spectra (**e-3,e-4**) correspond to cells before and after laser irradiation for 30 min at the intensity of 2.4 × 10^6^ W/cm^2^, respectively.

To visualize both the distribution of the SWCNTs inside the cancer cells and their agglomeration rate we employed G-band (1590 cm^−1^) and D-band (1330 cm^−1^) of the SWCNT Raman spectrum. The G-band was used to map the SWCNT distribution in the cytoplasm of the cell. Typical images of cells after their exposure to SWCNTs for 24 h and a typical Raman spectrum of SWCNTs accumulated in the cells are presented in Fig. 2. It was revealed that SWCNTs were accumulated inside rat C6 glioma cells in the form of micron-sized agglomerates, SWCNTs did not enter into the nuclei and remained only in the cytoplasm.

In our experimental conditions, the excitation of the Raman signal with a continuous laser beam at λ_ex_ = 785 nm did not lead to any observable cell destruction. The laser beam was focused on the largest SWCNT agglomerate inside the cells and the cell was exposed to laser irradiation (λ_ex_ = 785 nm, 7.2 × 10^3^ W/cm^2^, laser spot diameter 2 µm) for 10 min. Re-scan did not show any changes in the state of the cell membrane or the distribution of agglomerates of SWCNTs inside the cell after radiation exposure, as can be seen from Fig. 2a,b. No significant change was observed in the Raman spectra of the SWCNT agglomerate used for irradiation (see Fig.  2e-1(before irradiation) and 2e-2 (after irradiation)). Increasing of the laser intensity up to 2.4 × 10^6^ W/cm^2^ and reduction of the scanning step from 2 µm down to 0.5 µm that led to an increase in the scanning time and exposure time of the cell to laser radiation, improved spatial resolution to detect the presence of smaller SWCNT agglomerates (Fig. 2c,d), which were not visualized in the Fig. 2a,b. Nevertheless, a change in the scanning parameters of the sample did not lead to the cell membrane damage and any suppression of its viability.

The same cell was exposed to laser irradiation (λ_ex_ = 785 nm, 2.4 × 10^6^ W/cm^2^, exposure time 30 min, laser spot diameter 2 µm). An increase in intensity and exposure time did not have any effect on cell viability (Fig. 2c,d). Moreover, based on the analysis of the Raman spectrum of CNTs, it could be argued that there was no significant local heating of SWCNTs caused by the conversion of absorbed laser radiation into heat, as there was no increase in the D mode^40^ as compared to the spectrum of SWCNTs before irradiation (Fig. 2e 1–4).

**Figure 3:**
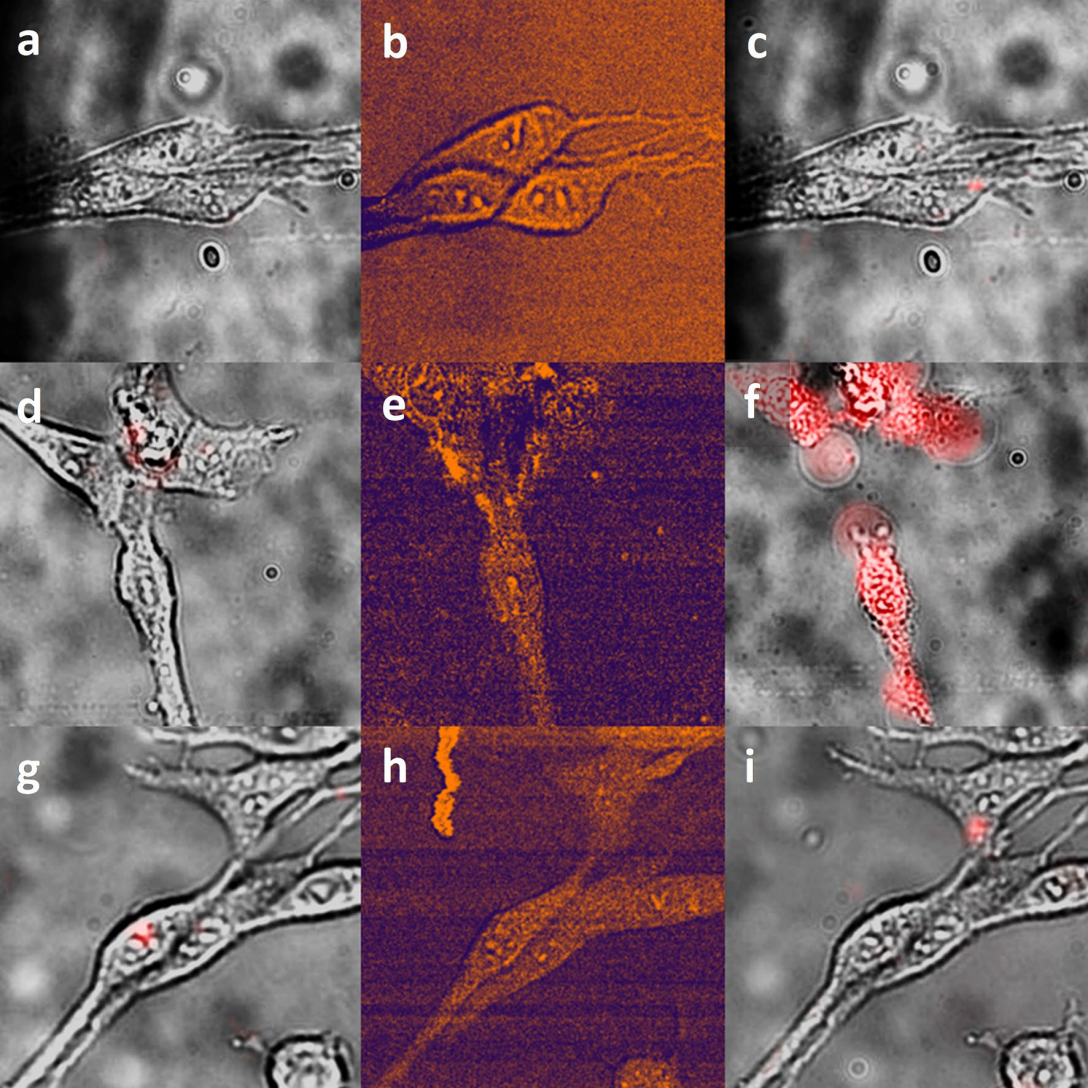
Photo-induced SWCNT-mediated destruction of glioma cells by NIR pico-second pulsed irradiation. (**a**–**c**) Bare C6 glioma cells; (**d**–**f**) C6 glioma cells with accumulated micron-sized SWCNT agglomerates, (**g**–**i**) C6 glioma cells in the presence of the SWCNTs suspension in the extracellular medium. (**a**,**d**,**g**) bright-field images superimposed with PI fluorescence images before irradiation; (**b**,**e**,**h**) CARS images; (**c**,**f**,**i**) bright-field images superimposed with PI fluorescence images after irradiation with 10 ps laser pulses (910.5/1064 nm, 10^6^ W/cm^2^) for 7 min.

Ultra-short laser pulses of high intensity can cause cell damage due to protein coagulation, vaporization, or photoablation^41^. Cells, cultured without SWCNTs, were exposed to picosecond laser irradiation (λ_ex_ = 910.5/1064 nm). The same laser source was used for simultaneous fast cancer cell visualization by coherent anti-Stokes Raman spectroscopic (CARS) imaging technique (Fig. 3b) performed at 1585 cm^−1^ wavenumber (C = C bending mode in proteins^42^). To exclude the possible harmful influence of picosecond laser pulses having an intensity of 10^6^ W/cm^2^^43^, cell viability was determined before (Fig. 3a) and after (Fig. 3c) the irradiation using fluorescent probe propidium iodide (PI). Exposure of glioma cells to irradiation for approximately 7 min (time for scanning 100 µm × 100 µm area) did not cause any plasma membrane permeabilization, as no PI fluorescence was detected (Fig. 3c).

Using CARS and fluorescent microscopy techniques we demonstrate that glioma cell plasma membrane stays intact and non-permeabilized after being irradiated with 10 ps pulses at λ_ex_ = 910.5/1064 nm and intensity of 10^6^ W/cm^2^. That is, these pulses do not have any destructive effect on the cells and can potentially be used for antitumor therapy and diagnosis.

Glioma cells cultured in the presence of SWCNTs for 24 h effectively accumulated SWCNTs intracellularly, and small SWCNT agglomerates were formed (see micro-Raman spectra in Fig. 2). It was shown, that exposure of cells to SWCNTs for 24 h and their accumulation inside the cell did not influence their viability. No PI fluorescence was detected on the cell membrane surface or somewhere in the external medium and growth surface of a Petri dish in the sample with glioma cells accumulated SWCNTs (Fig. 3d).These data indicate, that all the SWCNTs, detected by Raman microscopy (Fig. 2a–d), were located inside the cell rather than on the membrane^26^, and did not adhere to the plastic surface of the Petri dish, which could happen at the cell cultivation stage.

However, the exposition of the cells for 7 min with 10 ps laser pulses at the intensity of 10^6^ W/cm^2^ and 1 MHz pulse frequency (λ_ex_ = 910.5/1064 nm) led to plasma membrane permeabilization and pore formation, detected simultaneously with irradiation by CARS imaging (Fig. 3e). The CARS signal from cells decreased, but early stages of the violation of the integrity of the cell membrane were visualized. Afterwards, using fluorescent microscopy, violations of the integrity of the cell membrane and its death were demonstrated (Fig. 3f).

To evaluate the importance of the SWCNT intracellular accumulation step, NIR pico-second pulsed light interaction with SWCNTs dispersed in the extracellular medium of cells was investigated. Cells, grown without SWCNTs, but exposed to the medium, containing 5 µg/mL SWCNTs, stayed viable before and after the irradiation with pico-second laser pulses for 7 min because the glioma cell plasma membrane was not permeabilized (Fig. 3g–i).

Therefore our experiment shows that accumulation of SWCNTs inside the cell cytoplasm appeared to be crucial in photo-induced cancer cell destruction by pico-second NIR irradiation. Pulsed irradiation of cells with intracellularly accumulated SWCNTs led to the breaks in cellular membranes and cell death, detected both by CARS and fluorescent microscopy (Fig. 3e–f). Importantly, CARS enables for simultaneous irradiation and real-time visualization of cancer cells.

Simulation of pulsed irradiation interaction with SWCNTs’ micron-sized agglomerates and individually dispersed nanotubes fully proved this hypothesis. It was shown that absorption of irradiation by micron-sized agglomerate leads to photoacoustic destruction of glioma cells due to the propagation of the acoustic wave in the NP environment as shown in Fig. S.1b. If the negative pressure (volumetric extension) in the wave exceeds the threshold of ∆*P*_thresh_ ≈—0.7 MPa, destruction of the medium continuity may occur and lead to the destruction of the membrane integrity of the hosting cell. The developed model shows that laser pulses used in our experiment are capable of achieving the medium continuity destruction threshold. At the same time, local heating in the NP proximity is several degrees (see Supplementary Figure S.1a), which suggests that the photoacoustic mechanism of biostructure damage in this situation should prevail.

The simulation of the interaction of individual SWCNTs with laser pulses also fully supports our experimental results (Fig. 3g–i for experiment and Supplementary Figure S.2 for simulation). The amplitude of the generated pressure wave is six orders of magnitude lower than that generated in the SWCNT agglomerate. This finding allows us to conclude that in our experimental conditions, the probability of damage to the biostructures due to the generation of acoustic waves by solitary carbon nanotubes is negligible.

## Conclusions

We demonstrate that glioma cells are capable of efficient accumulation of SWCNTs to relatively large agglomerates, mainly inside the formed phagolysosomes. These agglomerates enable photo-induced destruction of cancer cells using picosecond laser irradiation. It is worth noting that neither bare cancer cell nor cells with SWCNTs dispersed extracellularly are not damaged under the same irradiation conditions. We also demonstrate that CARS can be effectively used for both visualization of the cancer cell and its photo-induced SWCNT-conditioned destruction, providing strong evidence that this method is a powerful tool for nanotheranostics. The advantage of using stable SWCNT suspensions instead of conventional NPs for photoacoustic therapy is the following: in the case of NPs, not only the cell accumulated NPs will be destroyed, but also normal tissues containing these NPs associated with tumor cells will be damaged. In the case of stable SWCNT suspensions, only cells that have accumulated SWCNTs as agglomerates are destroyed. Individual nanotubes in the extracellular environment are photoacoustically inactive. Therefore, there is no damaging effect on adjacent fluids and tissues.

The performed numerical simulation of the interaction of intense laser pulses with SWCNTs-enriched tissue may be employed to establish a common ground for the comparative study of the scattered literature data, to open a way toward increasing the efficiency of photo-induced CNT-conditioned destruction of tumors, and to reduce the cytotoxic load on the body during anticancer therapy.

## Methods

### SWCNT-DNA complexes

Single-walled CNTs produced by the HiPCO method (Nanointegris Technology Inc., Batch PO568) in bundles, purified from metal impurities (> 98%) were used throughout all the experiments. SWCNTs diameter varied from 0.8 nm to 1.4 nm (average 1.0 nm). Metallic to semiconducting nanotubes ratio was 1:2. SWCNTs were shortened down to 100–500 nm in length as described in^20^ and functionalized by salmon DNA (Sigma-Aldrich, USA). To obtain a DNA solution, 2 mg of the salmon DNA was placed in 6 ml of physiological solution for 2 days at a temperature of 4° C. Then the solution was treated with ultrasonic disperser UZDN-2 T for 30 min at 25% of the maximum power. Then, SWCNT agglomerates were added to 4 ml of the DNA solution and dispersed for 4–6 h at low ultrasonic intensity and room temperature. The resulting suspension was centrifuged for 10 min at 5000* g*. In this case, SWCNT agglomerates or SWCNTs not coated with a surfactant precipitated. The supernatant was centrifuged again to remove insufficiently dispersed material. The resulting SWCNT-DNA suspension was further used for biological applications. SWCNT-DNA (SWCNTs) concentration was determined via ultraviolet–visible spectroscopy (RV2201 spectrophotometer, ZAO SOLAR, Belarus)^44^. SWCNTs in suspension were stable for up to 4 months. Before each experiment SWCNTs in suspension were additionally centrifuged to remove large agglomerates formed in the suspension.

### Cell culture

C6 rat glioma cells (ATCC, CCL-107) were obtained from ATCC, LGC Standards (Ogrodowa 27/29, Kielpin, Poland) and grown in DMEM/F12 (Gibco, USA) medium supplemented with 10% newborn calf serum (Capricorn, Germany) and 80 µg/mL gentamycin sulfate (BelMedPreparaty, Belarus) at 37 °C, 100% humidity, and 5% CO_2_. For Raman spectroscopic studies 10^5^ cells were seeded per each Petri dish on silicon wafers and grown according to standard protocol. When cells were attached to the silicon surface, SWCNT suspension was added at a final concentration of 5 µg/mL. SWCNT accumulation and distribution inside the cells was investigated after 18–24 h of cells growing in the presence of SWCNTs. For fluorescent microscopy and light exposure studies, cells were seeded at low density (10^4^ cells per channel) in 6 channel slide (sticky-Slide VI 0.4, Ibidi, Martinsried, Germany) with a glass coverslip as a substrate. For all experiments we used external Hepes buffer solution of the following composition (in mM): NaCl—126, KCl—3, MgSO_4_—2, CaCl_2_—2, Hepes—10, and glucose—6.

### Raman spectroscopy of SWCNT distribution inside the cell

For Raman measurements, cells were seeded per each Petri dish on silicon (1 cm × 1 cm) to avoid strong background Raman signal from the glass. After 18 or 24 h incubation with SWCNTs, the cellular monolayer on the slide was washed twice with PBS. Raman spectra of the cell samples were measured using Raman confocal 3D-scanning microscope NanoFinder HighEnd (Lotis TII, Belarus—Tokyo Instruments, Japan) at the excitation wavelength of λ_ex_ = 785 nm (with a maximum power of about 75 mW). Exposition time was 0.5–2 s, the scanning step was 0.5–2.0 μm. Data are presented using the software packages of OriginPro 8.0 (OriginLab Corp., USA), Microsoft Excel 2007, NanoFinderViewer (Lotis TII, Belarus).

### Cells viability determination

To determine cell viability, propidium iodide (PI) as a fluorescent marker of cell membrane permeability was used. PI is a membrane-impermeable weakly fluorescent agent until it binds to DNA or RNA, causing 20–30 increase in fluorescence and 30–40 nm red shift of fluorescence excitation maximum at λ_ex_ = 535 nm and 15 nm blue shift of fluorescence emission maximum at λ_em_ = 617 nm^45^. When the cell plasma membrane is not intact and thermally induced pores appear, PI intercalates between the DNA bases inside the cell and strong fluorescence is detected. We used PI at the final concentration of 10^−6^ M in the external buffer solution throughout experiments and replaced PI-containing medium by Hepes buffer without PI washing the cells with continuous fluid flow.

SWCNTs in suspension were added to 2 channels in the 6 channel slide at final concentration 5 µg/mL and cells were cultivated in the presence of these complexes for 24 h before experiments. The other channels contained DMEM/F12 with supplements, used for routine culture procedure.

**Figure 4:**
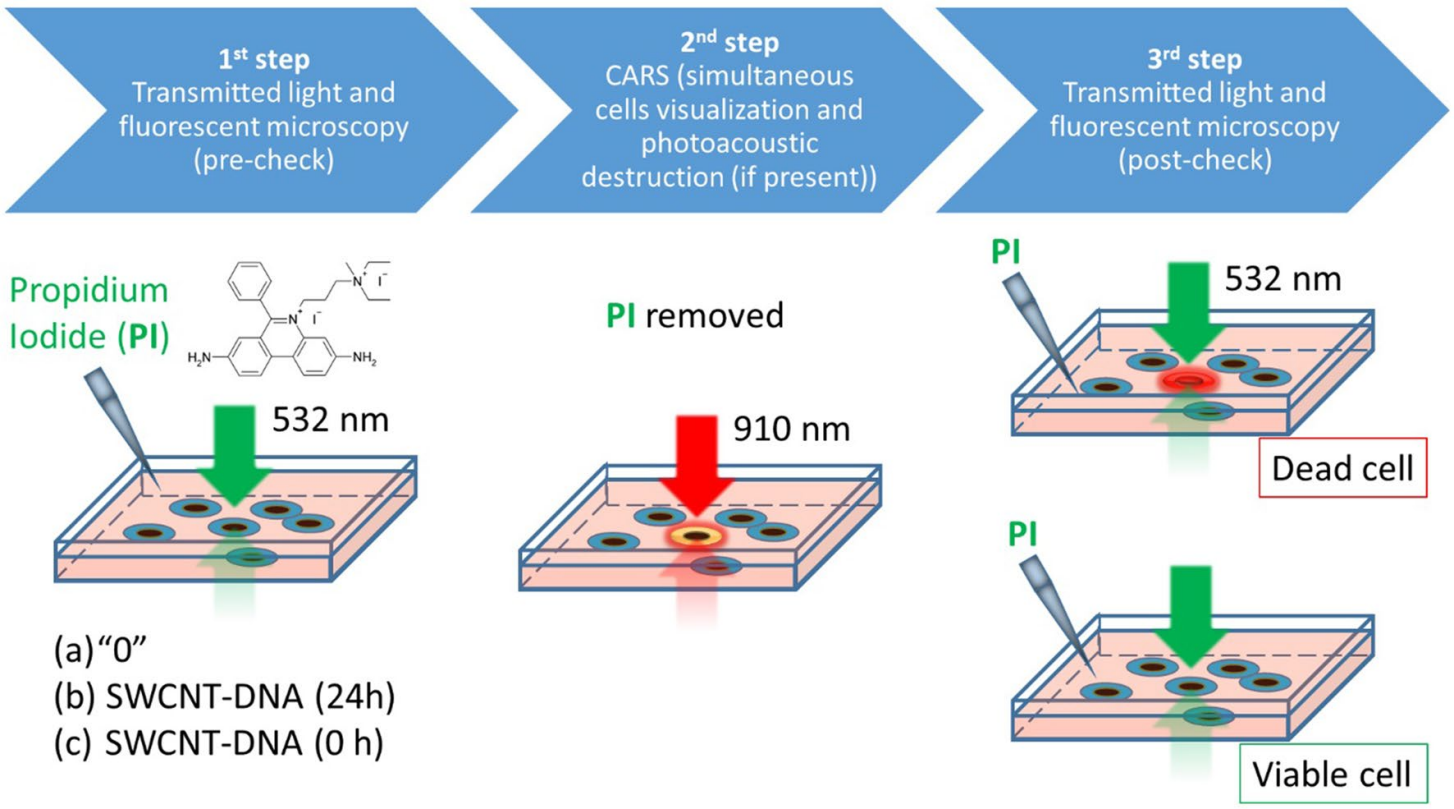
Experimental scheme for determination of glioma cell viability after NIR pulsed irradiation. (**a**) Cells without SWCNTs (control), (**b**) cells accumulated SWCNTs in the cytoplasm as micro-sized agglomerates, (**c**) cells exposed to stable suspension of individual SWCNTs.

Cells were irradiated under the microscope using continuous 785 nm laser irradiation of variable light doses on the 3rd day after seeding. For intense pulsed light treatment, a laser diode emitting a series of picosecond pulses was used. Cell samples were divided into three groups: cells without SWCNT-DNA complexes, cells exposed to SWCNT-DNA complexes for 24 h and accumulated SWCNTs inside the cells, and cells grown without SWCNT-DNA complexes, but irradiated in the external medium containing a high dose of SWCNTs (see Fig. 4).

### Coherent anti-Stokes Raman spectroscopy (CARS)

Home-made CARS system was used. Picosecond 1 MHz laser source (EKSPLA Ltd.) with dual-wavelength was combined with Microscope Olympus IX71 and a piezo scanning system (P-517.3CL, Physik Instrumente GmbH & Co) and utilized for raster-scanning of the sample. The exciting light was focused on the sample with an oil-immersion objective (Olympus, Plan Apochrom., 60X, NA 1.42). For detection of CARS signal the avalanche photodiode (SPCM-AQRH-14, Perkin Elmer), connected to a multifunctional PCI board (7833R, National Instruments) was used. Fundamental wavelength (1064 nm) and tunable-wavelength radiation (700–1000 nm) were used as Stokes and Pump excitation beams, respectively. Scanning of samples was performed at 1585 cm^−1^ wavenumber. For this, the pump beam was tuned to 910.5 nm and the resulting CARS signal within the spectral window from 840 to 782 nm was detected. Long-pass (cut-off at 860 nm) and short-pass (cut-off at 780 nm) filters were applied to spectrally separate the CARS signal. Excitation powers of 0.4 mW and 5 mW were employed for Pump and Stokes beams, respectively. Cells visualization was performed using the following set of parameters: laser-pulse duration 10 ps, intensity 10^6^ W/cm^2^, pulse frequency 1 MHz, scanning step 0.2 μm, acquisition time 1/500 s/pixel. The time for scanning the area of 100 × 100 μm using this set of parameters was 7 min.

### Fluorescence microscopy

The fluorescence images were acquired as in^46^. In short, we used a custom-built fluorescence microscope based on a commercial inverted biological Nikon Eclipse Ti-U with a 40 × CFI S Plan Fluor ELWD air objective and operated in the wide-field epi-illumination mode. The 532 nm CW DPSS laser (Crystalaser) was used as the excitation source. The diameter of the excitation spot was about 200 µm. The laser power after the objective was 0.6 mW. The excitation laser was directed into the objective by a 50/50 beam splitter. The resulting fluorescence was filtered off the excitation light with the long-pass filter (HQ545LP, Chroma) and imaged with EMCCD (DU-897E-CS0-UVB, Andor). The detector integration time was 0.1 s and each image was an average of 20 frames.

## Supplementary Information

Supplementary Information
