# Supplementary material for: Single-walled carbon nanotubes as a photo-thermo-acoustic cancer theranostic agent: theory and proof of the concept experiment
Source: Sci Rep. 2020 Dec 17;10:22174. doi: 10.1038/s41598-020-79238-6 (PMC7746693; doi:10.1038/s41598-020-79238-6)
Supplement: Supplementary file 1 — Supplementary Information [file 41598_2020_79238_MOESM1_ESM.pdf]

Supplementary information

Single-walled carbon nanotubes as a photo-thermo-acoustic cancer theranostics agent: theory and proof of the concept experiment

L. Golubewa [1,2,*], I. Timoshchenko [3], O. Romanov [3], R. Karpicz [1], T. Kulahava [2,3], D. Rutkauskas [1], M. Shuba [2,4], A. Dementjev [1], Yu. Svirko [5], P. Kuzhir [2,5]

[1] Center for Physical Sciences and Technology, Sauletekio Ave. 3, LT-10257 Vilnius, Lithuania

[2] Institute for Nuclear Problems of Belarusian State University, Bobruiskaya 11, 220006 Minsk, Belarus

[3] Belarusian State University, Nezavisimosti Ave. 4, 220030 Minsk, Belarus

[4] Tomsk State University, Lenin Ave. 36, 634050 Tomsk, Russia

[5] Institute of Photonics, University of Eastern Finland, Yliopistokatu 2, FI-80100 Joensuu, Finland

*Correspondence and requests for materials should be addressed to L.G. (lena.golubewa@ftmc.lt)

ORCID ID https://orcid.org/0000-0003-2125-6366.

Theoretical model and numerical simulation of the interaction of a picosecond laser pulse with tissue embedded with SWCNTs

1. Theoretical model

We model a living tissue with embedded nanoparticles as a homogeneous medium with inclusions having physical properties (absorption capacity, density, etc.) significantly differ from those of the surrounding medium. By assuming that the concentration of inclusions is low, we can ignore correlation effects and will study the light-induced temperature and acoustic perturbations associated with a single inclusion. We will consider the cylindrical and spherical inclusions (individual and agglomerated SWCNTs, respectively).

The photothermal effect in the medium containing nanoinclusions can be described by solving the following equations [1, 2, 3]:

– continuity equation: $$V = V_o\left(\frac{R}{r}\right)^{\alpha-1}\frac{\partial R}{\partial r};\qquad \text{(S1)}$$

– equation of motion: $$\frac{\partial u}{\partial t} = -V_0\left(\frac{R}{r}\right)^{\alpha-1}\frac{\partial P}{\partial r};\qquad \text{(S2)}$$

– Eulerian coordinate change equation R: $$\frac{\partial R}{\partial t} = u;\qquad \text{(S3)}$$

– equation of state: $$P = P(V, \varepsilon).\qquad \text{(S4)}$$

where V_o, V are initial and current specific volumes, respectively, r and R are Lagrangian and Eulerian coordinates, respectively, P is the pressure and ε are the specific internal energy. $\alpha = 2$ and $\alpha = 3$ correspond to the cylindrical and spherical nanoinclusions, respectively.

We approximate the equation of state by the Mie-Grünheisen equation in its binomial form [4]:

$$P = \rho_0 u_0^2\left(1 - \frac{V}{V_0}\right) + \Gamma\frac{C_V(T-T_0)}{V}\ ,\qquad \text{(S5)}$$

where $\rho_0 = 1/V_o$ is density, $\Gamma = \frac{u_0^2\boldsymbol{\beta}}{C_V}$ is the Grünheisen coefficient, β is volume expansion coefficient, C_V is the heat capacity at constant volume, u_0 is the speed of sound in the medium.

The temperature in the medium is described by the heat equation:

$$\rho C_V\frac{\partial T}{\partial t} = k_T\frac{1}{r^{\alpha-1}}\frac{\partial}{\partial r}\left(r^{\alpha-1}\frac{\partial T}{\partial r}\right) + Q_s,\qquad \text{(S6)}$$

where k_T is the thermal conductivity of the medium, $Q_s = k_{abs}I(r,t)$, where k_{abs} is the medium absorption coefficient, $I(r,t) = I_0 f_t(t) f_r(r)$ is light beam intensity at the time t at a point in space with a coordinate r, $f_t(t) = t/t_p e^{-t/t_p}$, where t_p is laser pulse duration, describes the temporal profile of the laser pulse. $f_r(r) = \begin{cases}1, r \le R_0\\ 0, r > R_0\end{cases}$, describes the spatial distribution of the heat source. R_0 corresponds either to the radius of the SWNT or radius of the spherical agglomerate of the SWNTs. Thus, we assume that the laser pulse energy is released in the whole volume of the inclusion.

The numerical solution of Eqs. (S1-S6) by a finite-difference approximation [5] allows us to obtain the spatiotemporal distribution of the pressure, temperature, density, and flow velocity in the inclusion and surrounding medium. It is worth noting that at the femtosecond excitation ($t_p < 10^{-13}\ s$), the two-temperature model can be employed for describing photothermal properties of liquid with metal inclusions [1]. The result of numerical simulation for the spherical SWCNTs bundles in the living tissue is performed in the main text in Fig.1 and allows us to reveal the conditions when the photothermal, photothermoacoustic and photoacoustic effects occur in the tissue embedded with the SWCNTs for a wide range of laser intensities and pulse durations.

The numerical simulation for the star in Fig.1c in the main text reflecting the experimental conditions was performed for the laser pulse duration of $t_p = 10^{-11}$ s at the intensity of $I_0 = 10^6$ W/cm^2. To model the agglomerated nanotubes, the spherical ($\alpha = 3$ in Eqs. (S1, S2)) inclusion radius was $R_0 = 1$ µm, density $\rho_0 = 1.357$ g/cm^3 [6], sound velocity $u_0 = 3 \cdot 10^6$ cm/s [7], heat capacity $C_V = 0.7$ J/g·K [8], thermal conductivity coefficient $k_T = 20$ W/cm·K [9], Grünheisen coefficient $\Gamma = 1.24$, light absorption coefficient $k_{abs} = 2.4 \cdot 10^5$ cm^{-1} [7]. To model individual nanotubes, we consider cylindrical inclusion ($\alpha = 2$ in Eqs. (S1, S2)) having a radius of $R_0 = 1$ nm. For the surrounding medium the following parameters were selected: density $\rho_0 = 1.025$ g/cm^3 [6], sound velocity $u_0 = 1.5 \cdot 10^5$ cm/s (taken equal to water), thermal conductivity coefficient $k_T = 0.45 \cdot 10^{-2}$ W/cm·K [6], heat capacity $C_V = 4.18$ J/g·K, Grünheisen coefficient $\Gamma = 2$.

2. Simulation results

The calculated light-induced pressure $\Delta P(r,t)$, temperature increase $\Delta T(r,t)$, and velocity $u(r,t)$ of the viscous medium with immersed spherical SWCNT agglomerate are shown in Fig. S.1 for the laser pulse duration of 10 ps. One can see that heating the inner area of the agglomerate results in moving of particles out from the center of the sphere ($u(r,t) > 0$) (Fig. S.1c, curves 1-4) forming the compression wave ($\Delta P > 0$, Fig. S.1b, curves 1-4). This wave is generated due to local heating ΔT (Fig. S.1a, curves 1-4) and the pressure gradient at the sphere surface (Fig. S.1b, curves 1-4). Then the wave reflects from the sphere surface ($u(r,t) < 0$, Fig. S.1c, curve 5) forming the negative component of the pressure wave

($\Delta P < 0$, Fig. S.1b, curve 5). Moreover, the minimum pressure values (in the negative phase) are achieved in the center of the sphere ($r = 0$). Further, the process of wave propagation inside the particle is repeated.

Due to the interband electron transitions, the absorption cross-section of CNTs has maxima in the NIR range. Exact match to resonant absorption allows achieving a maximal value of the absorption coefficient k_{abs}. The source term in equation S6 is $Q_s = k_{abs} I(r, t)$. An increase in the absorption coefficient is equivalent to the same intensity increase. So, if one wants to know how an increase in the absorption results in pressure modification, it is possible to find it in Fig. 1 (the main text of the manuscript) at the intensity increased to the same extent.

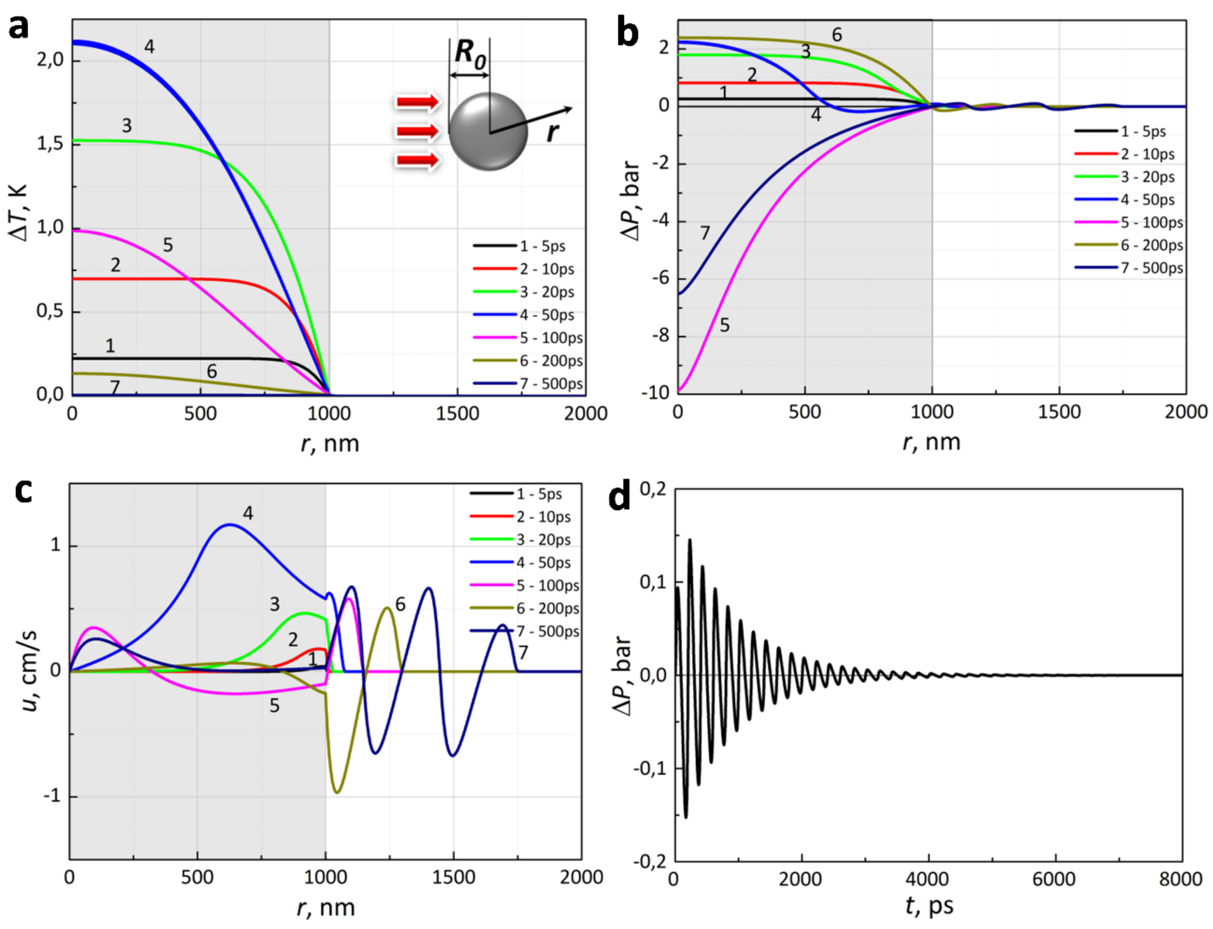

Figure S.1. Distribution of temperature (a), pressure (b), velocity (c) at various points in time (r is Lagrangian coordinate), and (d) time-dependent change in pressure in the environment of the absorbing particle (agglomerate of SWCNTs, $R_0 = 1$ μm).

Light absorption by an individual SWCNT can be modelled by the change of the temperature and pressure in a cylinder having a radius of $R_0 = 1$ nm. Figure S.2 shows the simulation results for the same parameters of the laser pulse, the absorption cross-section, and the environment as in the previous case. However, since the size of the absorbing particle is smaller than that in the previous case, a single SWCNT absorbs much less energy than the SWCNT agglomerate. Correspondingly, one can observe that change of the temperature and pressure is six orders lower in magnitude than in the previous case and cannot destroy the cancer cells. We also note that during the laser pulse, not only heating of the absorbing particle occurs as in the case of spherical agglomerate, but also heating of its environment due to the heat conduction mechanism (Fig. S.2a), which significantly reduces the pressure gradient between the heated and unheated regions (Fig. S.2b).

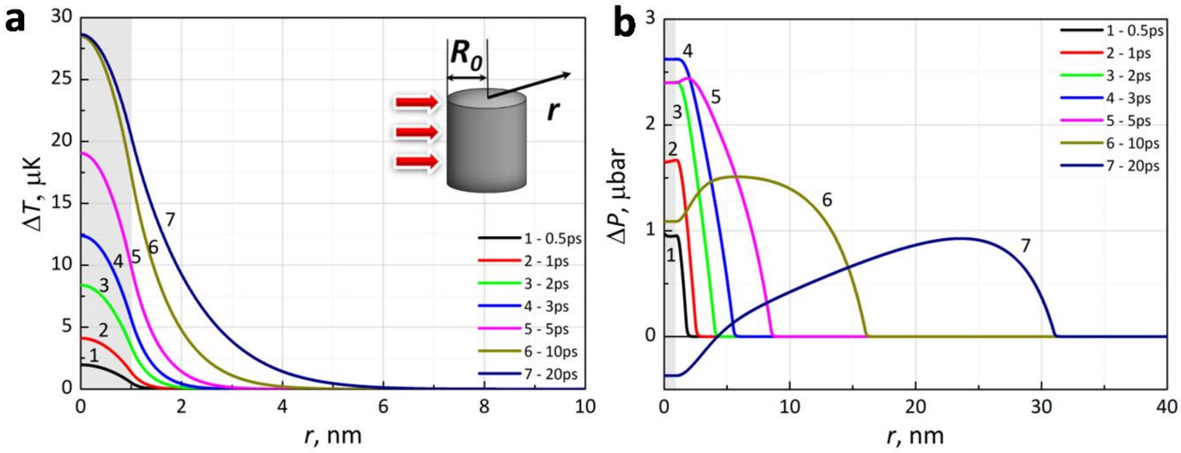

Figure S.2. Distribution of temperature (a) and pressure (b) at various points in time in the environment of absorbing particle (single-walled carbon nanotube, R_0 = 1 nm).

However, it has been numerously demonstrated experimentally that VIS-NIR irradiation of the cancer cells embedded with SWCNT has a considerable therapeutic effect, which is often explained in terms of the cell overheating [10] although such an explanation is not confirmed by the results of numerical modelling [11]. The performed analysis allows us to conclude that the observed therapeutic effect, which was erroneously described as photothermal, is due to photoacoustic and/or photothermoacoustic mechanisms depending on the parameters of the studied system.

References

1. Romanov, O. G., Zheltov, G. I. & Romanov, G. S. Action of femtosecond laser pulses on metal nanoparticles in a liquid. *Bull. Russ. Acad. Sci. Phys.* **75**, 1589–1591 (2011).

2. Zel'dovich, Y. B. & Raizer, Y. P. *Physics of Shock Waves and High-Temperature Hydrodynamic Phenomena*. (Dover Publications, Inc., 2002).

3. Richtmyer, R. D. & Morton, K. W. *Difference Methods for Initial Value Problems (Tracts in Pure & Applied Mathematics)*. (Intersci. Publ. div. John Wiley and Sons, New York, 1967).

4. Kanel, G. I., Razorenov, S. V., Utkin, A. V. & Fortov, V. E. *Shock-wave phenomena in condensed media [in Russian]*. (Yanus-K Publ., 1996).

5. Romanov, O. G., Zheltov, G. I. & Romanov, G. S. Numerical modeling of thermomechanical processes in absorption of laser radiation in spatially inhomogeneous media. *J. Eng. Phys. Thermophys.* **84**, 772–780 (2011).

6. Gong, F. *et al.* Effective thermal transport properties in multiphase biological systems containing carbon nanomaterials. *RSC Adv.* **7**, 13615–13622 (2017).

7. Haque, M. S., Marinelli, C., Udrea, F. & MilneW.I. Absorption characteristics of single wall carbon nanotubes. *Proc. NSTI Nanotechol. Conf.* **1**, 134–137 (2006).

8. Inoue, Y. *et al.* Anisotropic carbon nanotube papers fabricated from multiwalled carbon nanotube webs. *Carbon N. Y.* **49**, 2437–2443 (2011).

9. Pop, E., Mann, D., Wang, Q., Goodson, K. & Dai, H. Thermal Conductance of an Individual Single-Wall Carbon Nanotube above Room Temperature. *Nano Lett.* **6**, 96–100 (2006).

10. Sobhani, Z., Behnam, M. A., Emami, F., Dehghanian, A. & Jamhiri, I. Photothermal therapy of melanoma tumor using multiwalled carbon nanotubes. *Int. J. Nanomedicine* **12**, 4509–4517 (2017).

11. Siregar, S., Oktamuliani, S. & Saijo, Y. A theoretical model of laser heating carbon nanotubes. *Nanomaterials* **8**, (2018).
